# Eating for 3.8 × 10^13^: Examining the Impact of Diet and Nutrition on the Microbiota-Gut-Brain Axis Through the Lens of Microbial Endocrinology

**DOI:** 10.3389/fendo.2018.00796

**Published:** 2019-01-29

**Authors:** Joshua M. Lyte

**Affiliations:** Poultry Production and Product Safety Research Unit, Agricultural Research Service, United States Department of Agriculture, Fayetteville, AR, United States

**Keywords:** microbial endocrinology, diet, nutrition, microbiota-gut-brain axis, food, neuroendocrine, bacteria, probiotic

## Abstract

The study of host-microbe neuroendocrine crosstalk, termed microbial endocrinology, suggests the impact of diet on host health and microbial viability is, in part, reliant upon nutritional modulation of shared host-microbe neuroendocrine axes. In the 1990's it was first recognized that neuroendocrine pathways are major components of the microbiota-gut-brain axis, and that diet-induced changes in the gut microbiota were correlated with changes in host behavior and cognition. A causative link, however, between nutritional-induced shifts in microbiota composition and change in host behavior has yet to be fully elucidated. Substrates found in food which are utilized by bacteria in the production of microbial-derived neurochemicals, which are structurally identical to those made by the host, likely represent a microbial endocrinology-based route by which the microbiota causally influence the host and microbial community dynamics via diet. For example, food safety is strongly impacted by the microbial production of biogenic amines. While microbial-produced tyramine found in cheese can elicit hypertensive crises, microorganisms which are common inhabitants of the human intestinal tract can convert L-histidine found in common foodstuffs to histamine and thereby precipitate allergic reactions. Hence, there is substantial evidence suggesting a microbial endocrinology-based role by which the gastrointestinal microbiota can utilize host dietary components to produce neuroactive molecules that causally impact the host. Conversely, little is known regarding the reverse scenario whereby nutrition-mediated changes in host neuroendocrine production affect microbial viability, composition, and/or function. Mechanisms in the direction of brain-to-gut, such as how host production of catecholamines drives diverse changes in microbial growth and functionality within the gut, require greater examination considering well-known nutritional effects on host stress physiology. As dietary intake mediates changes in host stress, such as the effects of caffeine on the hypothalamic-pituitary-adrenal axis, it is likely that nutrition can impact host neuroendocrine production to affect the microbiota. Likewise, the plasticity of the microbiota to changes in host diet has been hypothesized to drive microbial regulation of host food preference via a host-microbe feedback loop. This review will focus on food as concerns microbial endocrinology with emphasis given to nutrition as a mediator of host-microbe bi-directional neuroendocrine crosstalk and its impact on microbial viability and host health.

## Introduction

Food composition underscores diet-induced changes in host health and behavior as well as the viability, composition, and functionality of the microbiome (1). Although the importance of the macro- and micro-nutrient content of food in driving such changes independently in host and microbiome is widely-appreciated, significantly less is understood regarding how food-induced changes in the microbiome mediated via evolutionarily shared neurochemistry can causally impact the host and vice-versa (2). Microbial endocrinology, which is the evolutionary-based study of the neuroendocrine routes of bi-directional communication between host and microbiome, has been proposed as a testable framework within which mechanistic pathways of the microbiota-gut-brain axis can be identified (3). It is important to recognize that several species of the microbiota share many of the same enzymatic pathways used by the host in the production of neurotransmitters and hormones. Food can contain, and is independently metabolized by host and microbe into, neuroendocrine molecules many of which (i.e., acetylcholine, serotonin (5-hydroxytryptamine; 5-HT), gamma-amino butyric acid (GABA), histamine and others) are *structurally identical* regardless of host or microbial origin. As both host and microbe express many of the same receptors with which to recognize these molecules (4), the neuroendocrine axes constitute an evolutionary-based, bi-directional interkingdom language. Seminal experiments in 1992 were the first to demonstrate that microorganisms directly respond to neuroendocrine hormones (5). Since that time, microbial endocrinological-based mechanisms have been hypothesized to play a critical role by which the microbiome influences host food preference (6) and appetite (7). Indeed, microbes produce key food intake-regulatory hormones, such as somatostatin (8), as well as affect host ghrelin, leptin, insulin, glucagon-like peptide (GLP)-1, and other neuroendocrine molecules (9).

Although it is well-recognized that host diet causes alterations in the microbiome (10), little attention has been given to how food-induced changes in host physiology may affect the microbiome. Specifically, as the microbiota-gut-brain axis is bi-directional, microbial endocrinological-mechanisms involve gut-to-brain as well as brain-to-gut pathways. Food contains numerous non-nutritive substances that influence host physiology and behavior. For example, caffeine, a psychoactive found in coffee, tea, as well as some foods can elicit a response by host neuroendocrine stress pathways (11) known to interact with the microbiota. Multiple other feedback loops exist between food-induced changes in host and microbe. In addition to psychoactive substances, commonly consumed food and beverages have been known for decades to contain neurotransmitters of plant or microbial origin. Plant sources of animal feed, such as silage, contain histamine and food for human consumption, as an example, tea can contain GABA. Histamine in silage (12) is also detectable in cattle feces (13), which suggests microbiome exposure to neurotransmitters of diet origin. Further, this evolutionary-based crosstalk based on shared neurochemistry has also been observed in farm production animals where it has been shown to affect ruminant eating behavior (14). Likewise, in human volunteers, GABA intake (15) has been demonstrated to reduce fatigue (16) and psychological stress (17).

Such neurotransmitter crosstalk between host, food and microbe also represents a means by which food-induced changes in the microbiome can causally impact the host. Indeed, bacteria that inhabit the gastrointestinal tract (18) are capable of utilizing host dietary elements in the biosynthesis of neuroendocrine molecules, such as dopamine (19, 20). Human fecal isolates of several bacterial genera produce biogenic amines (21) which affect host health. For example, *Morganella morganii*, which produces histamine from amino acids and is found in the human gastrointestinal tract, is one of several bacterial taxa responsible for the production of histamine in spoiled fish, the consumption of which can cause anaphylactic shock and death (22). Hence, diet provides precursors useable by the microbiome in the *de novo* production of signaling molecules that can affect host neuroendocrine axes.

Many aspects of diet, including food composition, consumption patterns, and cultural habits therefore have the potential to affect host-microbe interaction via diverse neuroendocrine routes involving the microbiota-gut-brain axis (Box 1). Microbial endocrinology stands to provide a strong conceptual framework for the design of testable hypotheses in the pursuit of uncovering mechanisms by which diet and nutrition mediate changes in the host or microbiome along the shared evolutionary bridge of neuroendocrine communication.

Box 1 **Current knowledge and future research directions.**

**What is known?**

- Diet contains a wide range of neuroendocrine molecules and their precursors (2, 23).
- Many of the neuroendocrine constituents in foods survive the digestive process and are absorbed in the upper gastrointestinal tract or reach the gut lumen (24, 25).
- Oral intake of neuroendocrine molecules has been reported to affect cognitive and emotional outcomes in volunteers and animals (16, 26, 27).
- The ability of nutrition to alter the microbiota and influence the microbiota-gut-brain axis and thereby influence memory and learning has been shown (28).
- Several members of the microbiota in a variety of host species have been demonstrated to respond to, uptake, and synthesize neuroendocrine molecules which are structurally-identical to those produced by the host (3).
- Certain products found in foods, such as caffeine, have direct effects on host stress neuroendocrine axes (29).
- Stress is well-recognized to influence eating behavior and food choice (30); and many foods, including chocolate (31), contain neuroactive components which can affect host emotional and cognitive state.

**What is unknown?**

- Does the neuroendocrine content of food affect compositional, and more importantly, functional changes in the microbiome? Would such changes be meaningful to the host? Could a diet be designed to specifically feed the microbiota with therapeutic implications for the host?
- Stress neuroendocrine axes are hubs of host-microbe crosstalk. How do foods that directly affect host stress physiology, such as caffeine and the hypothalamic-pituitary-adrenal (HPA)-axis, alter host-microbe interaction?
- How do legal requirements and cultural preferences in food processing (e.g., pasteurized vs. unpasteurized dairy products), as well as geographical considerations such as soil composition and crop cultivation, create population-specific exposure to neuroendocrine content in food?
- Does host-microbe neuroendocrine crosstalk influence host stress-induced feeding behavior, such as comfort eating, and disorders such as binge-eating?
- Although the ingestion of neuroendocrine-rich foods such as banana can increase postprandial plasma catecholamine concentrations, what, if any, are the implications of such changes on the host? Moreover, as catecholamines in the bloodstream can reach the gut, what, if any, are the implications on the microbiota and host-microbe interaction?

## Overview of Review Structure

**Figure 1:**
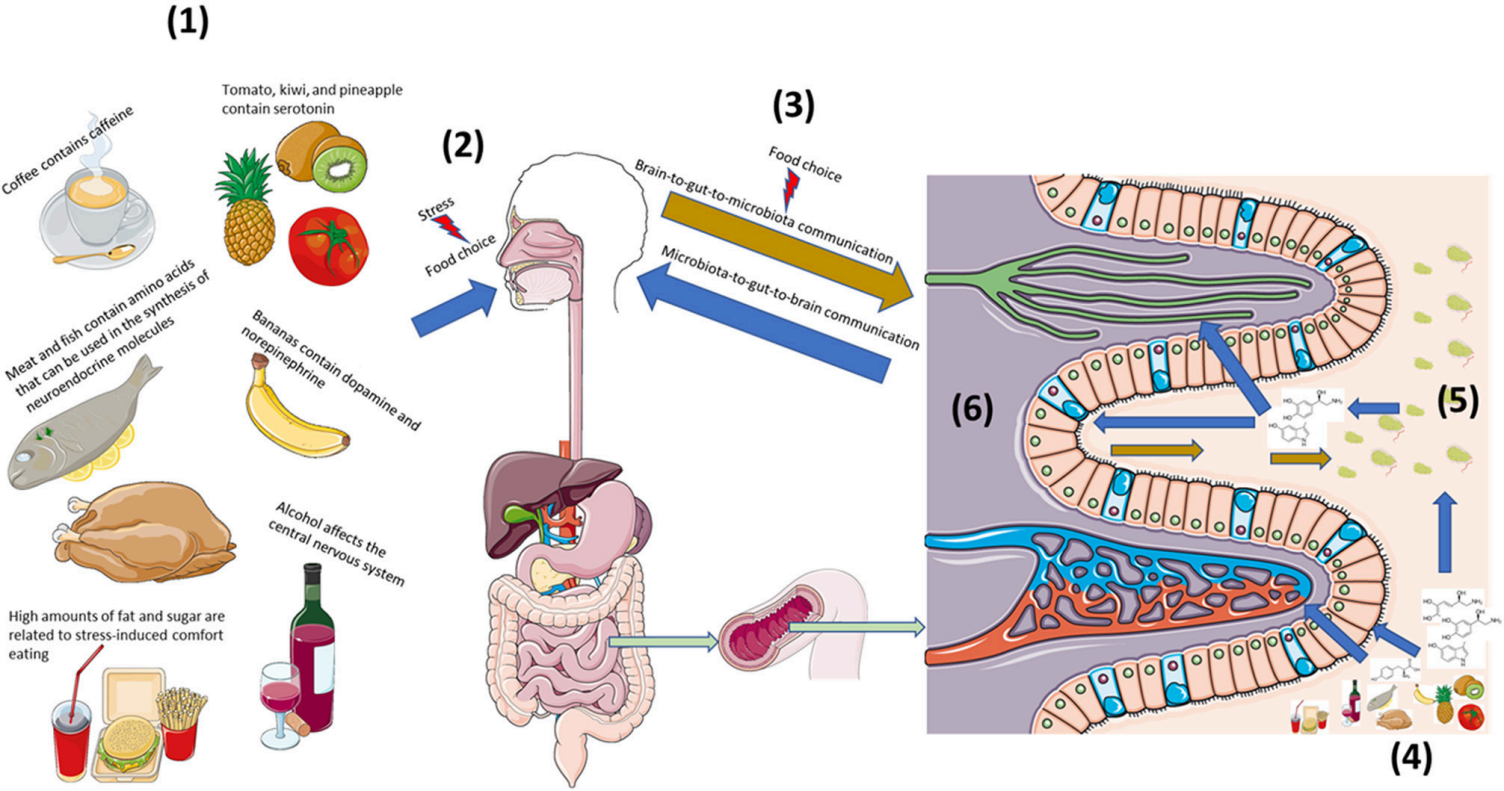
Diet strongly influences bi-directional neuroendocrine crosstalk along the microbiota-gut-brain axis. (1) Diet is both a source of neuroactive and neuroendocrine molecules as well precursors that can affect host neuroendocrine physiology (2). (2) Stress can influence food preference and consumption, (3) which can affect host-microbe bi-directional communication. (4) Neuroendocrine constituents and precursors found in foods act locally on host intestinal cells as well as the (5) microbiota to influence local interkingdom crosstalk. (6) Capillaries, lymphatic channels, as well as afferent and efferent nerves can extend into gut villi to provide multiple routes of bi-directional communication along the microbiota-gut-brain axis. This figure was made using templates adapted from Servier Medical Art by Servier. Original images are licensed under a Creative Commons Attribution 3.0 Unported License; https://smart.servier.com.

In examining the role of diet on the bi-directional neuroendocrine pathways that underscore host-microbe crosstalk via the microbiota-gut-brain axis, it is necessary to draw from diverse literatures such as food and agricultural sciences, microbiology, endocrinology, clinical psychiatry and neuroscience as well as others. This review will first address the presence of neurochemicals and relevant precursors in food and then progress to discussing how these can affect known neuroendocrine axes of host-microbe interaction. From there, food-induced changes in the microbiota-gut-brain axis will be examined from bottom-up (i.e., microbiota-to-gut-to-brain) and top-down (i.e., brain-to-gut-to-microbiota) perspectives (Figure 1).

## Neuroendocrine Molecules and Precursors Found in Food

As neuroendocrine pathways play significant and diverse roles in plant, mammalian, avian, aquatic life, insect, and reptilian physiologies, it should not come as a surprise that diet is a source of neuroendocrine molecules and their precursors (Table 1). It should be emphasized that this is not exclusive to human diets but includes many types of livestock animal feeds, as well as the diets of wildlife. This section will serve to give a brief overview of select neuroendocrine content of common food sources [for a comprehensive overview, the reader is directed to a recent review (23)] with associated outcomes related to their consumption. Due to the many types of digestive systems ranging from humans to wildlife to insects, it is not possible to generalize which neuroendocrine components and precursors survive digestion.

**Table 1:** A non-exhaustive summary of the neuroendocrine and neuroendocrine-precursor content of common foods.

**Food**	**Neuroendocrine molecule /precursor**	**Reference(s)**
**FRUIT**		
Banana	Serotonin; Dopamine; Norepinephrine	(32, (33)
Kiwi	Serotonin	(25)
Pineapple	Serotonin	(25)
Tomato	Serotonin	(25)
Avocado	Dopamine	(34)
Orange	Dopamine	(34)
**VEGETABLE**		
Spinach	Serotonin; GABA,	(35, 36)
Potato	GABA	(37)
Green onion	Serotonin	(35)
Eggplant	GABA; Acetylcholine	(38)
Broccoli	Dopamine	(34)
**LEGUME**		
Fermented soybean products	Histamine, tyramine, other biogenic amines	(39)
**MEAT/FISH**		
Cured meats	Histamine, tyramine, other biogenic amines	(40)
Aged meats	Altered amino acid profiles compared to fresh meat	(41)
Beef	Tyrosine, other amino acids	(42)
Chicken	Tyrosine, other amino acids	(43)
Pork and ham	Tyrosine, other amino acids	(44)
Fish	Histidine, tyrosine, other amino acids	(45)
**DAIRY**		
Cheeses	Histamine, tyramine, other biogenic amines	(46, 47)
Fermented milks	Biogenic amines	(48)
**SEEDS/NUTS**		
Coffee bean	Serotonin	(49)
Cocoa bean	Dopamine	(34)
Hazelnut	Serotonin	(50)
Walnut	Dopamine; Serotonin	(51, 52)
Pecan	Serotonin	(53)

Dietary amino acids tryptophan, tyrosine, and histidine to name a few, are precursors to the neurotransmitters serotonin, dopamine/norepinephrine/epinephrine, and histamine, res-pectively. A wide range of protein sources serve as rich sources of these amino acids including—but not limited to—fish, meat, legumes, nuts, and cheeses. Tryptophan, an essential amino acid in humans, is commonly employed in psychiatric-based clinical and preclinical studies to assess the relationship between serotonergic function and neuropsychiatric diseases such as depression. Acute tryptophan depletion in animals and humans, achieved through diet, has helped demonstrate the multifactorial relationship between serotonergic function and neuropsychiatric disease (54). Tyrosine, a non-essential amino acid in humans which can be obtained through diet, has been shown to enhance resilience to cognitive impairment during stress (55, 56), reduce environmental stress (57), as well as elevate anger under stressful conditions (58). Histidine, an essential amino acid in humans, is found in significant quantities in fermented products such as dried bonito flakes. Human consumption of dried bonito flakes has been associated with improved mood and reduced fatigue (59). Oral ingestion of histidine was demonstrated to improve working memory and attentiveness in healthy volunteers (60) and animals (61).

Like amino acids, neuroendocrine molecules and other precursors found in foods can survive the digestive process to reach the intestinal tract. Despite abundant evidence of neuroendocrine communication between host and microbe, surprisingly few microbiota-gut-brain axis-focused publications have examined the consequence of consuming neuroendocrine-rich foods. Indeed, the majority of investigations examining the fate and effect of dietary-derived neuroendocrine molecules have been published in the clinical and neuropsychiatric-based literature.

GABA is a constituent of several edible plants including those used in the preparation of tea (62). Investigations examining the effects of oral administration of GABA have shown GABA can be readily absorbed by the gastrointestinal tract, appearing in the plasma 1–1.5 h postprandial (24). *Ex vivo* and *in vitro* studies of animal (63) and human (64) intestinal tissues have identified potential GABA transport mechanisms. It is important to note that GABA receptors within the enteric nervous system are widely distributed throughout the gastrointestinal tract (65) and that GABA plays several physiological roles in a region-dependent manner along the gastrointestinal tract. Additionally, afferent ends of the vagal nerve, a pathway of gut-to-brain communication (66), extend into villi of the small intestine (67) and express GABA receptors (68, 69). Although GABA supplementation has been reported as effective in mediating favorable behavioral outcomes in animals and people, the mechanisms by which this occurs remain largely unknown as it is unclear whether, and under what conditions, GABA may cross the blood brain barrier (BBB) (70). Hence, it is unknown whether reports demonstrating that oral administration of GABA results in a reduction of fatigue in animals (27) and human volunteers (16), as well as antidepressant-like effects (26), improved task completion (71), and anti-insomnia effects (72) (73), are due to GABA acting locally on gastrointestinal receptors in a putative gut-to-brain mechanism, are due to circulating GABA, or if some GABA does indeed cross the BBB.

Serotonin is found in significant quantities in bananas, and a potential clinical role for serotonin obtained through banana consumption to treat constipation in human subjects was tested decades ago (33). Although it is controversial whether serotonin affects gastrointestinal motility and fecal transit (74), dietary-derived serotonin does reach the intestinal tract, is absorbed and undergoes hepatic metabolism into 5-hydroxyindoleacetic acid (5-HIAA) which can result in a postprandial elevation in plasma 5-HIAA concentration (75) and excretion in the urine (76). Other dietary sources of serotonin, such as nuts, pineapple, tomato, and kiwi have been shown to cause unique changes in human postprandial serum concentrations of 5-HIAA compared to banana (25).

Bananas also contain abundant amounts of dopamine and norepinephrine (32). Healthy volunteer plasma catecholamine concentrations were elevated following banana consumption (77). Likewise, urinary concentrations of homovanillic acid, a major metabolite of dopamine, increased after having eaten bananas (34). Banana, as well as other dietary sources of catecholamines such as pineapple and walnuts, were also reported to affect plasma concentrations in healthy volunteers of multiple catecholamine metabolites, including normetanephrine and 3-methoxytyramine which are breakdown products of norepinephrine and dopamine, respectively (78).

Although the Cavendish banana, a major commercial cultivar (79), has high concentrations of monoamines, a comparison of monoamine levels of other cultivars of banana consumed throughout world is needed. Cultural (i.e., governmental regulations and food safety laws) as well as geographic (i.e., soil, climate, and other conditions that affect, for example, crop growth) influences likely shape population-specific exposure, and therefore health and economic implications, to unique neuroendocrine profiles of common foods. For example, biogenic amine concentrations are typically lower in pasteurized compared to unpasteurized dairy products as pasteurization reduces microbial load, including bacteria that produce biogenic amines (46). The consumption of dietary-derived biogenic amines can have direct effects on host physiology. For example, consumption of tyramine-rich (tyramine etymology stems from the words tyrosine and amine, “tyros” is Greek for “cheese”) foods is contraindicated for patients prescribed monoamine oxidase inhibitors (MAO-I) in the treatment of psychiatric illness (80). Typically, dietary tyramine, which survives stomach acid, is metabolized within the intestine by monoamine oxidase (MAO). The use of MAO-I inhibits MAO which allows excess tyramine to enter circulation. Once present in the circulatory system it can then be converted to the catecholamine norepinephrine, thereby resulting in the precipitation of a hypertensive crisis.

Likewise, the geographical origin of food sources is well recognized to affect the chemical compositions of livestock feed ingredients (81, 82) which in turn can impact digestibility (83). Biogenic amines in animal feeds appear to have species-specific effects and are not strictly deleterious or beneficial (84). For example, poultry broilers fed diets that included high concentrations of putrescine, cadaverine, histamine, and/or phenylethylamine did not exhibit reduced performance or display histopathologic alterations (85). This literature review did not reveal any study that profiled the neuroendocrine content of different feeds sourced either domestically or internationally.

## Interaction of Neuroendocrine Axes and Food Intake—a Microbial Endocrinological Perspective

Meal timing and feeding behavior are intimately linked to neuroendocrine physiology. The sensitivity of circadian cellular and physiological functions to chrono-patterns of feed intake has been recognized for decades (86). However, such findings largely have yet to integrate recent discoveries of bacterial regulation of feeding behavior and influence on metabolically-important host neuroendocrine profiles. For example, ghrelin, an orexigenic hormone (87) that influences central dopamine (88) and serotonin (89), is increased in rodent plasma before scheduled meal times and changes in serum ghrelin concentrations have been correlated with higher or lower counts of certain fecal bacterial taxa (90). Likewise, germ-free mice exhibit reduced hypothalamic ghrelin concentrations which are normalized following colonization with a conventional microbiota (91). Nevertheless, ghrelin knockout rodents exhibit normal appetite and feed intake (92), suggesting compensatory mechanisms in driving food consumption. As ghrelin's orexigenic effects are mediated through dopaminergic signaling (93), and dopamine has been suggested to influence appetite (94), microbial utilization of the dopamine precursor L-dopa (95) may suggest a microbial endocrinological route of host appetite modulation. Hence, in a study that utilized human subjects, reduced appetite reported by *Helicobacter pylori*-infected patients (96) may be linked not only to lowered ghrelin via *H. pylori* effects on gastric endocrine cells, but to *H. pylori* utilization of L-dopa and the resulting impact on central dopaminergic function.

Other chronobiological pathways that regulate feeding behavior also are likely to include bi-directional microbial endocrinological-based mechanisms. Changes in leptin, an anorexigenic hormone, are subject to both circadian rhythm and patterns of feed intake. Although leptin is derived from gastric and adipose origin (97), gastric leptin is secreted into both the gastric juice as well as into the bloodstream (98). In addition to gastric leptin appearing in circulation, gastric leptin also reaches the intestinal lumen (99). As intestinal epithelial cells express leptin receptors on both the basolateral membrane and on the apical microvilli, gastric leptin exerts diverse effects on the intestinal epithelium (100). Little is known, however, regarding how leptin affects the gastrointestinal microbiome. Interestingly, it has been reported that, compared to conventional mice, germ-free mice exhibit greater leptin sensitivity within the central nervous system (CNS) (101). Although the authors did not speculate on a microbial-based mechanism being responsible for the relative leptin resistance observed in conventional mice, clues may still be gleaned from other studies. For example, leptin knockout mice display both hyperphagia and alterations in gastrointestinal microbial diversity (102). Conversely, knockout of murine intestinal epithelial leptin receptor, which was identified in the submucosa of each region of the small intestine, did not cause compositional changes in the fecal microbiome (103).

Thus, a key question not addressed in the literature, is whether leptin itself has any direct effects on the microbiota (i.e., do bacteria sense and uptake extracellular leptin?), especially given that gastric leptin reaches the gut lumen and interacts with the intestinal epithelium. Relatedly, *ob/ob* mice, which are obese due to hyperphagia from being leptin-deficient, exhibit increased intestinal bacterial translocation possibly related to dysregulated glucose metabolism (104) compared to conventional mice (105). When these *ob/ob* mice were administered leptin, intestinal mucosal bacterial adherence and translocation were reduced. Hence, leptin signaling may be a source of host-microbe neuroendocrine bidirectional communication. As the evidence indicates that leptin affects bacterial adherence and translocation in the intestine, it can therefore be suggested that leptin serves to eliminate unwanted microbial colonization of the gastrointestinal tract. If a direct inhibitory effect of leptin on certain members of the microbiota is found, perhaps bacterial tolerance to leptin may represent an evolutionary niche by which certain species are able to colonize different regions of the upper gastrointestinal tract, such as the jejunum (106). Indeed, it would be interesting to investigate whether leptin signaling, via a microbial endocrinological-based mechanism, unites the separate observations that obesity is associated with leptin resistance (107) as well as the risk of small intestinal bacterial overgrowth (108).

Whether host-microbe dialogue via neuroendocrine stress pathways mediates the effects of stress on dietary consumption patterns is a particularly relevant, yet little understood, intersection of feeding behavior and the microbiota-gut-brain axis. For example, stress is recognized to affect eating behavior (109), and while significant attention has been given to associations of the microbiota and changes in feeding behavior (i.e., “correlation”), very little is understood in the way of causation (110). The first evidence that neurohormones constitute an interkingdom language between prokaryote and eukaryote was reported in the early 1990s (5, 111, 112). Microbial endocrinological-based investigations have since revealed the mechanisms by which several stress-related neurohormones, including serotonin and norepinephrine allow for bi-directional communication between host and microbe. While the causation underlying stress-induced eating disorders is multifactorial, the involvement of central catecholaminergic pathways in feeding behavior has been recognized for decades (113). Likewise, vagotomy [i.e., severing of the vagus nerve, a major bi-directional route of microbiota-gut-brain communication 114] has been reported to associate with decreased hunger, gustatory intensity, and related sensations (115). Rodent studies have demonstrated the microbiota to significantly alter host catecholaminergic systems (116) and, for example, via the vagus nerve, affect brain function (117) as well as anxiety- (118) and depressive-like behaviors (119). It is important to note that several types of vagal afferents are distributed throughout the mucosa of different regions of the gastrointestinal tract (120), and that the receptors found on vagal afferents are not homogenously distributed; hence, functionality of vagal afferents cannot be generalized across different gastrointestinal regions (121). Nevertheless, some vagal afferents lie in close proximity to enteroendocrine cells (EEC) (122). As the apical surface of EEC can reach the gut lumen, EEC luminal sensing of microbial metabolites and dietary components allows for paracrine activation of receptors found on vagal afferents (123). Indeed, diet-induced changes in the microbiota were recently associated with alterations in the vagal afferent innervation of the rodent cecum (124). EECs express several types of receptors, including adreno- and serotonin-receptors. Application of norepinephrine to murine colonic intestinal epithelium *ex-vivo* was shown to elicit sensory neuronal response via an EEC-based mechanism (125). Likewise, in rodents, in response to luminal stimuli, serotonin released from intestinal EEC was demonstrated to activate the vagal afferent 5-HT_3_ receptor (126). Interestingly, the proximal colon contains vagal afferent endings which are mechanosensitive (127), and proximal colon distension activated catecholaminergic neurons in the brainstem (128). Since the brainstem plays a critical role in receiving gut-derived signals in order to regulate food intake (129), it is therefore reasonable to suggest that host-microbe neuroendocrine signaling within the gastrointestinal tract may signal via the vagus nerve to affect feeding-regulatory regions of the CNS.

In addition to vagal afferent innervation, the intestinal tract is innervated by vagal efferent nerves (130, 131). This is an important consideration as it allows the host to rapidly respond to signals, including those that are food-related, originating from the gut via vagal afferent stimulation. One such vagal afferent/efferent circuit is the cholinergic anti-inflammatory pathway (CAP) (132). Indeed, the effect of a high fat diet in reducing pro-inflammatory cytokine response in rats that underwent hemorrhagic shock was shown to be mediated via the CAP (133). Hence, enteric nutrition may hold therapeutic potential in mediating inflammatory response to trauma via a gut-to-brain vagal mechanism. It is important to note that it is unknown how CAP activation may differ depending on intake of different types of dietary fat, or whether other dietary-acquired constituents such as 5-HT may act on vagal afferent receptors in gut villi to initiate CAP.

## Microbial Influences on Host Feeding Behaviors: From Microbiota-to-Gut-to-Brain

“I'm cuckoo for Cocoa Puffs!®”—Sonny the Cuckoo Bird®

Although it may never be known whether microbes influence Sonny's® predilection for Cocoa Puffs®, several lines of evidence suggest the microbiome can interact with host feeding-related physiology, food preference, as well as affect food-seeking behavior and cognition. Indeed, recent studies have described a role for the microbiome in influencing host intake and chemo-sensing systems of carbohydrate (134), fat (135), and protein (136). The first evidence linking diet-induced changes in the microbiome to alterations in host behavior and cognition was reported in 2009 (28). Compared to mice maintained on a standard chow diet, mice fed a meat-based diet exhibited both significantly greater fecal microbial diversity which was positively correlated with improvements in working and reference memories, as well as comparatively less anxiety-like behavior and a slower speed in seeking food. Little is understood, however, of how such diet-induced shifts in the microbiota mechanistically affect the host. From within the gastrointestinal tract, microbes can utilize host dietary components in the *de novo* synthesis of neuroendocrine molecules and precursors that may affect host health and hedonistic behaviors via microbial endocrinological-pathways of the gut-brain axis (2).

EEC-mediated mechanisms represent one such pathway by which the microbiota can potentially affect the host through diet via microbial endocrinological-based signaling. EEC express a repertoire of luminal metabolite- and nutrient-sampling receptors. Such receptors include sweet-taste receptors which bind caloric sugars and non-nutritive sweeteners (137), bitter-taste receptors, free-fatty acid receptors (FFARs) which bind dietary fatty acids as well as microbial-generated short-chain fatty acids (138), and amino acid receptors (139). Compared to conventional rodents, germ-free rodents displayed greater preference for drinking solutions that contained higher concentrations of sucrose (134). Dietary sugars, such as sucrose in drinking water, are absorbed in the upper small intestine, including the duodenum and jejunum (140). Duodenal EEC-induced release of serotonin in response to maltose, a disaccharide of glucose, or glucose has also been shown to elicit duodenal vagal afferent firing (126). Relatedly, the microbiome composition was observed to diverge between mouse lines selectively bred for low or high saccharin taste phenotype (6). Low or high saccharin preference in rodents has been reported to be associated with social behavior as well as reactivity to stress (141). As the vagus nerve is important in mediating some probiotic anxiolytic effects on the host (3), it may be hypothesized that any microbial influence on host preference for the type and quantity of sugar intake could drive behavioral effects via an EEC-vagus-mediated mechanism of the microbiota-gut-brain axis.

Also found in the distal ileum and the large intestine, EEC can be exposed to products of bacterial metabolism of metabolic host dietary constituents that escaped absorption in the small intestine. Non-digestible carbohydrates, such as resistant starch, can be fermented by the microbiota into short chain fatty acids (SCFAs). Butyrate, one such microbial SCFA, can act on human EEC FFARs to stimulate the release of peptide YY (PYY) (142). In rodents, PYY, a satiety signal, has been shown to bind to the Y2 receptor on neighboring gut vagal afferents to activate hypothalamic neurons that help regulate food intake (143). Likewise, butyrate can influence host catecholamine synthesis (144) and, at certain concentrations, elicits activation of the hypothalamic-pituitary-adrenal (HPA)-axis, a classical neuroendocrine pathway of the stress response (145). As nerve terminals that ramify into the intestinal mucosa synthesize dopamine and norepinephrine (146), butyrate may act locally in the gut to alter luminal catecholamine production. As HPA-axis activation is associated with anxiety, it is important to note that mice fed *ad-libitum* high amylose type-2 resistant starch exhibited changes in the microbiota that associated with host anxiety-like behavior (147). Colonic epithelial cells (148) as well as endothelial cells of the BBB (149) both express the SCFA transporter monocarboxylate transporter (MCT)-1. Hence, butyrate and acetate may be transported from the intestinal lumen into the circulation to cross the BBB and thereby affect brain and behavior. For example, radio-labeled acetate absorbed from the colon can cross the BBB and suppress appetite in rodents (150). Multiple routes may therefore enable microbial SCFAs, derived from host dietary components, to potentially influence the host along neuroendocrine pathways of the microbiota-gut-brain axis.

The microbiota also affects the amino acid profiles of the gastrointestinal tract (151) and circulation. For example, intestinal microbial-produced free lysine and threonine has been detected in human plasma (152). Lysine was recently shown to play an important function in avian neuroendocrine regulation of food intake, however, a role for the microbiota was not investigated (153). Amino acids are precursors to several microbial-derived neuroendocrine molecules, including the biogenic amines agmatine, derived from lysine; cadaverine, derived from arginine; and putrescine, derived from ornithine (154). Each of these biogenic amines have been demonstrated to be related to diet. For example, the oral administration of agmatine in rats was reported to affect host metabolism accompanied by reductions in diet-associated weight gain (155). Likewise, mice fed a high protein diet had elevated cadaverine in colonic content compared to mice on a moderate protein intake (156). Finally, hypertensive obese patients fed a hypocaloric diet with cheese containing *Lactobacillus plantarum*, a probiotic which synthesizes putrescine, exhibited reduced body mass index (BMI) and arterial blood pressure compared to patients fed cheese without the probiotic (157).

The microbiota can also utilize host dietary amino acids in the synthesis of xenobiotic compounds, such as indoles, which affect host health and behavior (158). Microbial-produced indoles, which are derived from tryptophan, can affect host anxiety-like behavior (159). Nevertheless, what may be gleaned from these studies is that much remains to be understood regarding not only the extent of microbial production of the amino acid-derived neuroendocrine molecules in the gastrointestinal tract *in vivo*, but whether the *in vivo* production, and effect on the host, of these products mirrors what has been reported from their oral ingestion.

As mentioned earlier in this review, bananas contain significant concentrations of free catecholamines, which following banana consumption, appear as sulfate-conjugated catecholamines in the plasma of healthy volunteers (77). In this study, the authors suggested that “sulfate conjugation of free catecholamines in banana largely takes place in the gut.” Specifically, conjugation of free catecholamines takes place predominantly in the mesentery (160). The site of conjugation is important as microbial β-glucuronidase in the gut lumen was recently discovered as necessary for the deconjugation of sulfate- and glucuronide-conjugated catecholamines (116) as well as serotonin (161). Hence, within the gut lumen the microbiota plays a critical role in the conversion of biologically-inactive, conjugated monoamines to biologically-free, active monoamines. As the microbiota responds to free catecholamines with significant implications for host health (2) and, for example, uptake extracellular serotonin (4), it will be critically important to understand how the ingestion of neurochemical-containing food affects the microbiota to causally-impact the host. Moreover, as the microbiota can convert dietary amino acids into biologically-active monoamines in simulated *in vivo* conditions (20), it will be essential to determine how host food intake and dietary habits fuel microbial production of monoamines that can then affect neuroendocrine host-microbe crosstalk along the microbiota-gut-brain axis.

## Host Feeding Behavior Influences the Microbiota: From Brain-to-Gut-to-Microbiota

“They're Gr-r-r-reat!”—Tony the Tiger®

Tony the Tiger's® subjective opinion of Frosted Flakes® might or might not be shared with his microbiota. Indeed, food selection is well-recognized to be influenced by conditions of psychological and physical stress. Comfort eating, food choice which lessens the feelings of stress (30), is engaged in by both sexes as well as animals and has been shown to affect physiological response (162–164) and emotional perception (165) of stress. In light of recent findings in rodents that stress and sex uniquely interact to influence the microbiota-gut-brain axis (166), and that the microbiota can affect host food choice, it is important to note that the effect of comfort eating in reducing stress in female rodents is estrous-cycle dependent (167). Likewise, chemical constituents of foods, such as caffeine, can elicit HPA-axis activation. As food therefore can affect host neuroendocrine systems, this gives rise to altered brain-to-gut signaling which could affect the microbiota.

How foods influence host neuroendocrine physiology is multifactorial. Chocolate is a principal example (168) as it contains the psychoactive substances threonine and caffeine, a combination of fat and sugar, a subjectively-appealing aroma, in addition to other factors. Recent studies, however, have highlighted a role for the fat and sugar, perhaps the latter more so than the former, content of chocolate in being the main drivers of chocolate's psychoactive effects (31). Foods that contain high sugar and fat were shown to alter rodent stress-induced eating behavior (169). The effects of comfort eating on the HPA-axis are well-described, with glucocorticoids playing a major role in increasing the intake of palatable food (170). Glucocorticoids, including corticosterone, are synthesized by intestinal epithelial cells and released into the gut lumen (171). For example, fecal corticosterone can be measured in rodents (172), canines (173), and wildlife (174) as a non-invasive indicator of environmental stress. As glucocorticoids are released into the gut lumen, it is reasonable to assume the microbiota are exposed to host-produced glucocorticoids. An effect on microbial viability, growth, or function following exposure to corticosterone or other major host-produced glucocorticoids is unknown. Gnotobiotic animals, such as the defined 8-species microbiota of the Altered Schaedler Flora (ASF) rodent, may allow for interrogation of how host glucocorticoids or neuroendocrine molecules secreted into the gut affect the microbiota in a brain-to-gut-to-microbiota mechanism (175). Hence, deliberate host food choice under times of stress to reduce the host's own stress response may causally impact the microbiota.

Caffeine, a psychoactive substance found in coffee, tea, and other beverages and foods, is consumed in virtually all societies (176). Although health regulatory bodies, such as the European Food Safety Authority have set recommended daily caffeine intake limits for children and adults as 3 mg/kg and 400 mg (total), respectively, animal (11) and human studies (177, 178) have demonstrated caffeine as anxiogenic. Indeed, a single caffeine dose of 250 mg in healthy adult volunteers caused an elevation in anxiety and diastolic blood pressure (29). That low doses of caffeine alter the host HPA-axis is notable for several reasons. First, HPA-axis responsivity is part of the bi-directional microbiota-gut-brain axis (179). HPA-axis activation stimulates the production of the stress catecholamines norepinephrine and epinephrine that effect unique and direct responses in the microbiome (180, 181). As norepinephrine and epinephrine are found at several host-microbiome interfaces, including in the gut lumen (116, 125) and lung alveolar fluid (182), it is reasonable to suggest that changes in the host HPA-axis and its responsiveness following food or beverage consumption likely affect the microbiome.

Relatedly, in healthy subjects, consumption of a triple espresso or 250 mg of caffeine has been shown to stimulate sympathetic nervous system (SNS) activity (183). SNS activation can cause the release of catecholamines from the adrenal medulla, which feed back onto the HPA-axis, sympathetic nerve fibers also ramify throughout the intestinal tract. As sympathetic nerve terminals express tyrosine hydroxylase and synthesize norepinephrine, that they can be found in the intestinal mucosa (184) provides yet another route of neuroendocrine host-microbe crosstalk. Indeed, norepinephrine from sympathetic efferent nerve terminals throughout the body can escape breakdown to appear in the gut (185) as well as plasma (186). It is currently unknown whether the intake of caffeine or other substances in foods that activate the SNS may influence the microbiome.

Like caffeine, alcohol consumption is a virtually global occurrence (187). Alcohol affects the CNS as well as causes physical changes in several areas along the gastrointestinal tract (188). Protracted alcohol abuse can cause dramatic changes in CNS neurochemistry and brain structure (189). It is largely unknown how alcohol's direct degenerative effects on the CNS may bi-directionally impact the microbiota-gut-brain axis. Nevertheless, alterations in neuronal architecture, one may assume, would contribute to changes in the microbiota-gut-brain axis in the direction of the brain-to-gut-to-microbiota. This direction of the microbiota-gut-brain axis should be considered in the context of alcohol use, as alcohol can be absorbed in the stomach and upper small intestine (190), therefore not all alcohol ingested may reach the intestinal tract and microbiota. It should be noted that substantial attention has been given to alterations in the gut microbiota following mild as well as chronic alcohol consumption, and hypotheses have been proposed describing alcohol's influence on the microbiota-gut-brain axis in the direction of the microbiota-to-gut-to-brain (191, 192).

## Conclusions and Future Directions

The relationships between diet, nutrition, and the microbiota-gut-brain axis are incredibly complex, yet mechanistic pathways which causally unite these categories remain to be fully elucidated. We are truly at the beginning of our understanding of these complex inter-relationships. The neuroendocrine system is a major bi-directional set of pathways along the microbiota-gut-brain axis that is both affected by food choice and at the same time is a locus of host-microbe crosstalk. Microbial endocrinology, the study of neuroendocrine host-microbe interkingdom communication, represents an important lens, and certainly *not the only one*, through which diet, nutrition, and the microbiota-gut-brain axis may be mechanistically linked. Indeed, foods and beverages contain diverse neuroendocrine molecules and precursors that separately affect host physiology as well as microbial viability and function. Moreover, microbial utilization of host dietary components can result in the production of neuroendocrine signaling molecules that can affect host health, especially considering that several microbe-derived molecules are structurally identical to those produced by the host. Likewise, diet-induced changes in host neuroendocrine physiology likely have direct consequences for the microbiota. Microbial endocrinological-based mechanisms are a promising avenue by which future nutrition-oriented microbiota-gut-brain axis research may identify causal, mechanistic-driven pathways of how diet affects the bi-directional nature of host-microbiota interaction that ultimately may define host health and behavior.

## Author Contributions

The author confirms being the sole contributor of this work and has approved it for publication.

### Conflict of Interest Statement

The author declares that the research was conducted in the absence of any commercial or financial relationships that could be construed as a potential conflict of interest.
